# CRISPR/Cas9-Mediated Enrichment Coupled to Nanopore Sequencing Provides a Valuable Tool for the Precise Reconstruction of Large Genomic Target Regions

**DOI:** 10.3390/ijms24021076

**Published:** 2023-01-05

**Authors:** Giulia Lopatriello, Simone Maestri, Massimiliano Alfano, Roberto Papa, Valerio Di Vittori, Luca De Antoni, Elisa Bellucci, Alice Pieri, Elena Bitocchi, Massimo Delledonne, Marzia Rossato

**Affiliations:** 1 Department of Biotechnology, University of Verona, Strada Le Grazie 15, 37134 Verona, Italy; 2 Department of Agricultural, Food and Environmental Sciences, Polytechnic University of Marche, Via Brecce Bianche, 60131 Ancona, Italy; 3 Genartis srl, Via IV Novembre 24, 37126 Verona, Italy

**Keywords:** de novo assembly, variant calling, Cas9-tiling enrichment, nanopore sequencing, pod-shattering

## Abstract

Complete and accurate identification of genetic variants associated with specific phenotypes can be challenging when there is a high level of genomic divergence between individuals in a study and the corresponding reference genome. We have applied the Cas9-mediated enrichment coupled to nanopore sequencing to perform a targeted de novo assembly and accurately reconstruct a genomic region of interest. This approach was used to reconstruct a 250-kbp target region on chromosome 5 of the common bean genome (*Phaseolus vulgaris*) associated with the shattering phenotype. Comparing a non-shattering cultivar (Midas) with the reference genome revealed many single-nucleotide variants and structural variants in this region. We cut five 50-kbp tiled sub-regions of Midas genomic DNA using Cas9, followed by sequencing on a MinION device and de novo assembly, generating a single contig spanning the whole 250-kbp region. This assembly increased the number of Illumina reads mapping to genes in the region, improving their genotypability for downstream analysis. The Cas9 tiling approach for target enrichment and sequencing is a valuable alternative to whole-genome sequencing for the assembly of ultra-long regions of interest, improving the accuracy of downstream genotype–phenotype association analysis.

## 1. Introduction

The identification of genetic variants underlying phenotypic diversity in plants is central to many studies focusing on conservation and evolution, as well as pre-breeding programs for crop improvement. Genome-wide association studies (GWAS) support the systematic identification of candidate loci responsible for phenotypic variation. In plants, GWAS typically involves large panels of inbred lines derived from a variety displaying the phenotype of interest, from which genetic and phenotypic information is retrieved. When genotyping is carried out by resequencing, reads generated from each individual are usually mapped to the corresponding reference genome in order to identify single nucleotide variants (SNVs) and small insertions/deletions (indels). Statistical methods are then applied to identify the genomic loci associated with the desired phenotypes and/or to fine-map candidate genes and regions underlying them.

The strategy outlined above is hindered by the intrinsic limitations of the reference genome, affecting variant calling and the precise identification of genetic variations, with a significant impact on downstream analysis. First, an incomplete reference genome assembly may result in missing variability and/or in read-mapping artifacts, leading to false positive variants [1]. For example, the recent completion of the human reference genome improved variant calling sensitivity and reduced the number of false positives [2]. Second, reference genomes are usually generated from cultivated and highly homozygous lines (double haploids or inbred lines) whereas the donors of target phenotypes may be phylogenetically distant wild or undomesticated relatives [3,4]. The profound differences between such individuals and the reference genome may produce incorrect alignments, leading to variant miscalling [5]. Most importantly, different individuals, cultivars or landraces of the same species may feature consistent structural variations (SVs) and copy number variations (CNVs) that influence their phenotypes. Such genomic variations can result in profound changes to the sequence, structure and even copy number of genes, such that thousands of paralogs can be missing from the reference assembly [1,3,6,7,8].

A reference pangenome can be constructed to cover the entire genetic diversity of a species and this comprehensively accommodates genetic variations underlying specific phenotypes. However, pangenomes are available for only a few plant species [7,9,10,11,12]. The generation of pangenomes with structural information requires the availability of high-quality assemblies based on long-read sequencing, but the generation of numerous long-read assemblies per taxon can be cost-prohibitive, especially for species with large genomes. Targeted de novo assembly methods for the reconstruction of specific regions linked to a phenotype of interest would make the investigation of population-wide variability more efficient while maximizing the number of individuals that are analyzed and compared.

Target enrichment is increasingly applied in combination with long-read sequencing methods such as the Pacific Biosciences Single Molecule Real-Time (PacBio SMRT) and Oxford Nanopore Technologies (ONT) platforms. As well as reducing data costs, targeted sequencing accelerates downstream bioinformatics analysis by focusing solely on the target reads. Target enrichment has been achieved by long-range PCR or pull-down using biotinylated probes [10,13,14,15,16,17]. However, these approaches depend on PCR amplification, which suffers from GC-content bias and allelic dropout and can, therefore, lead to genotyping errors and other artifacts [1,18]. For the same reason, the size of the enriched region is usually limited to <10–15 kbp [17]. More recently, CRISPR/Cas9-based enrichment has emerged as a promising approach that is not dependent on amplification, allowing the enrichment of targets up to 80 kbp [19,20,21,22,23]. The enrichment of longer targets (up to 100 kbp) is possible using the ONT platform if shorter non-target fragments that compete for sequencing are depleted [24]. Longer targets can also be enriched using an approach known as Cas9 tiling, in which the target region of interest (ROI) is divided into smaller, overlapping sub-regions (sub-ROIs) that are enriched and sequenced separately. However, this is a relatively new approach and the largest sub-ROI that has been sequenced thus far is 25 kbp [25,26]. Even so, this approach was shown to resolve SNVs and SVs at the haplotype level, which was not possible with short reads [22,23]. The validity and accuracy of the method for de novo assembly of large ROIs compared to traditional long-read whole-genome sequencing (WGS) is not yet clear.

Here, in the framework of the INCREASE project [27], we assessed the potential of CRISPR/Cas9-mediated enrichment combined with ONT sequencing for the de novo assembly of a 250-kbp region on chromosome Pv05 of the common bean genome (*Phaseolus vulgaris*) associated with pod indehiscence [28,29,30,31]. The Cas9 tiling approach was adapted for the sequencing of ultra-long targets and used for the reconstruction of this region in the non-shattering *P. vulgaris* cultivar Midas. Cas9-based assembly identified several SNVs, indels and SVs in Midas compared to the reference genome, some of which affected genes. There was a high concordance with the results of WGS-derived reconstruction. We demonstrate that Cas9 tiling is a valuable, reliable and cost-effective alternative to traditional de novo assembly based on WGS for the reconstruction of large ROIs, facilitating downstream association and fine-mapping studies.

## 2. Results

The pod-shattering region diverges significantly between the Midas cultivar and the *P. vulgaris* reference genome.

**Figure 1:**
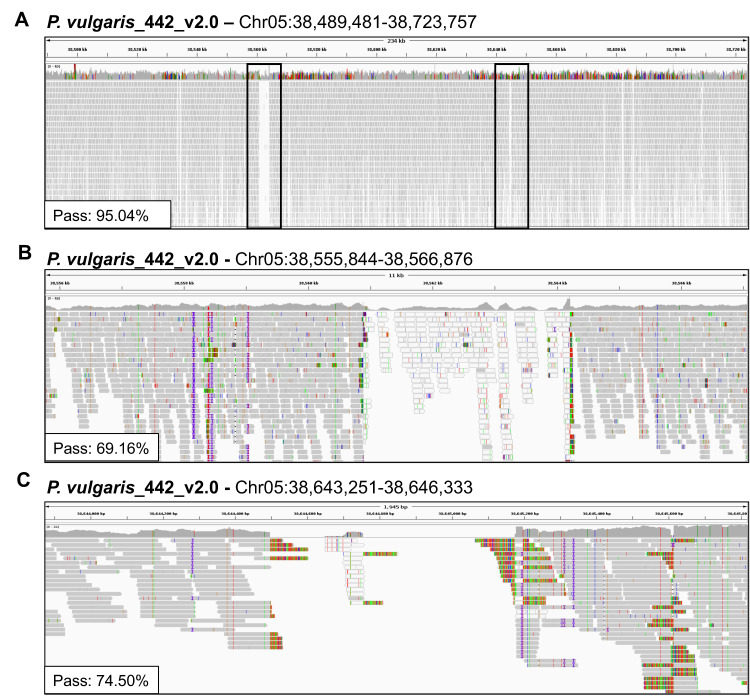
Sequencing data show high divergence between the Midas cultivar and the *P. vulgaris* reference genome within the pod-shattering region. (**A**) Integrative Genome Browser Visualization (IGV) of Midas Illumina whole-genome sequencing reads mapped to the pod-shattering region (Chr05:38,489,481–38,723,757). Alignments to the regions highlighted with black squares are magnified in panels (**B**,**C**) and represent the regions with the highest divergence. Each IGV shows the associated genotypability in the same region (% PASS at DP ≥ 5).

To identify genetic variants associated with the indehiscence phenotype we sequenced the Andean snap bean landrace Midas, which features non-shattering pods. Illumina short-read sequencing generated ~48 million fragments with 24.6× coverage of the *P. vulgaris* reference genome, but the quality of read alignment was poor in the pod-shattering region (Chr05:38,489,481–38,723,757) (Figure 1A). Genotypability, defined as the fraction of positions where a base can be called reliably (% PASS at ≥5 read depth) was 95% on average across the whole ROI but dropped to lower values in some sub-ROIs (Figure 1A–C). In particular, nine portions of the reference genome ranging in size from 195 to 3320 bp were either not covered or poorly covered (<5×) by sequencing data, or showed reads mapped with quality “zero” (Figure 1B,C). Moreover, reads mapping to these regions frequently showed clear soft-clipped portions, suggesting the presence of SVs in the Midas cultivar (Figure 1C). These results suggested that reconstruction of the pod-shattering region present is *P. vulgaris* reference genome does not resemble the corresponding region of the Midas cultivar.

### 2.1. Reconstruction of the Pod-Shattering Region by CRISPR/Cas9 Tiling and ONT Sequencing

To generate a contiguous assembly of the ROI in the Midas genome, we combined CRISPR/Cas9 tiling as an enrichment method with ONT sequencing (Figure S1A). First, we designed guide RNAs (gRNAs) matching the *P. vulgaris* reference genome and selected coding regions with no SNVs or indels based on Midas WGS Illumina data (Figure S2). The gRNAs were designed to cut five sub-ROIs, each of ~50 kbp, overlapping by ~2–3 kbp and covering the entire ROI (Figure S2). We split gRNA pairs into two cutting reactions carried out in parallel (Figure S2 and Table 1).

**Figure 2:**
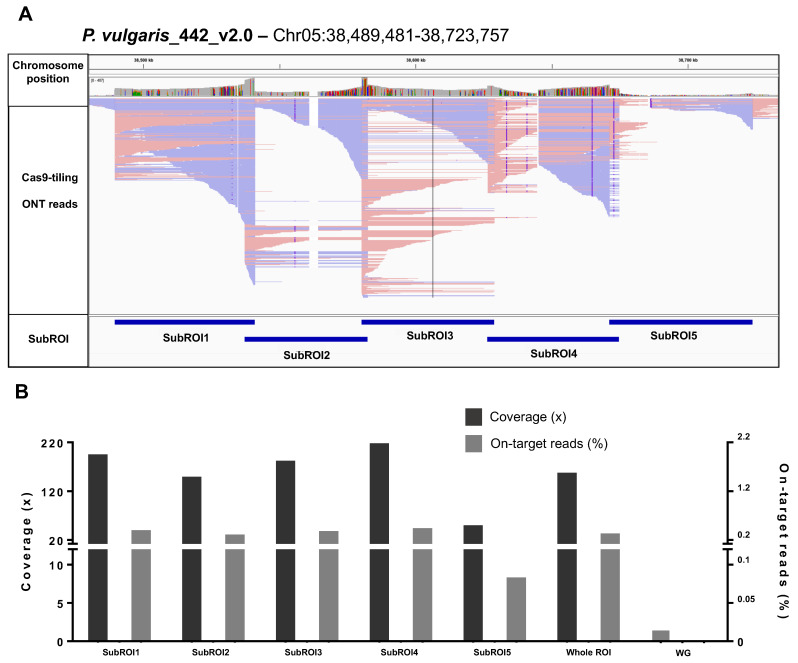
Sequencing of the pod-shattering region by combining Cas9 tiling with ONT sequencing. (**A**) Integrative Genome Browser Visualization (IGV) of ONT data mapping to the pod-shattering region (Chr05:38,489,481–38,723,757) after Cas9 tiling and ONT sequencing of Midas DNA. (**B**) Fraction of ONT reads and average coverage on each sub-ROI, the whole ROI, and whole genome (WG) after Cas9 tiling and ONT sequencing.

Cas9 tiling generated 157,028 ONT reads, of which 54,540 were PASS, with an N50 value of ~30 kbp (Table 1 and Figure 2A). Reads mapping to the *P. vulgaris* reference genome resulted in variable on-target read percentages and on-target average coverages across the different sub-ROIs, with sub-ROI4 showing the highest values and sub-ROI5 the lowest (Figure 2A,B). The whole target region was covered with a total of 1794 reads and the average coverage was 152.85× (Figure 2A,B). The de novo assembly of ONT reads generated a single contig of 229 kbp that was polished using Illumina WGS data from the Midas cultivar, followed by annotation as described in the methods section (Table 2).

**Figure 3:**
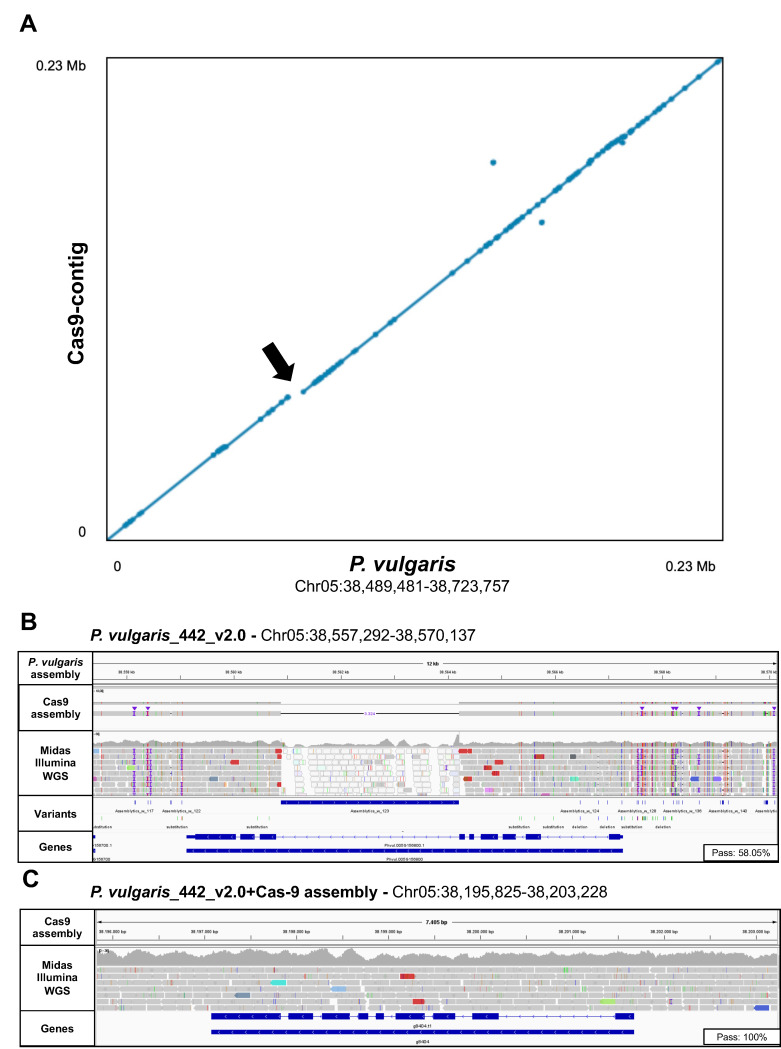
The pod-shattering region features multiple variations and a large deletion of 3 kbp overlapping a gene, when comparing the Midas cultivar and *P. vulgaris* reference genome. (**A**) The contig assembly based on Midas Cas9 tiling data (*y*-axis) was aligned to the pod-shattering region of the *P. vulgaris* reference genome (*x*-axis) using NUCmer. Alignments longer than 1 kbp were subsequently filtered and visualized with Dot viewer. Aligned dots highlight regions where the two sequences diverge, and the arrow shows a ~3-kbp deletion in Midas (magnified in panels (**B**,**C**)). (**B**) Integrative Genome Browser Visualization (IGV) of Cas9 assembly and Midas Illumina WGS reads aligned to the *P. vulgaris* reference genome (Chr05:38,557,292–38,570,137) revealing the detailed tracks of SNVs and SVs identified in the Cas9 assembly compared to the reference genome and the annotated gene overlapping the deletion marked in panel (**A**). (**C**) IGV of Midas Illumina WGS reads aligned to the Cas9 assembly (Chr05:38,195,825–38,203,228) in the region including the orthologous gene shown in panel **B**. Internal boxes show the genotypability (% PASS at ≥5 reads) of the two orthologous genes.

**Figure 4:**
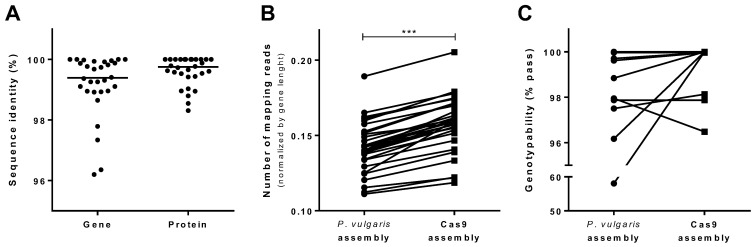
Comparison of sequences and mapped reads of orthologous genes in the *P. vulgaris* reference genome and Cas9 assemblies. (**A**) Percentage sequence identity of the orthologous genes and corresponding proteins derived from the *P. vulgaris* reference genome and Cas9 tiling assembly (excluding untranslated regions, which were not annotated de novo in the Cas9 assembly). (**B**) Number of Midas WGS Illumina reads mapping onto the orthologous genes from the *P. vulgaris* reference genome and Cas9 tiling assembly, after normalizing by each gene length. (**C**) Proportion of bases that can be genotyped (% PASS at ≥5 reads) in the orthologous genes from the *P. vulgaris* reference genome and Cas9 tiling assembly. *** *p*-value < 0.0001, paired *t*-test.

Comparison of the Cas9-assembled contig with the corresponding region of the *P. vulgaris* reference genome (Figure S1A) showed a large number of variations, namely 1163 SNVs, 288 insertions and 318 deletions, of which 427 (36.7%), 50 (17.4%) and 56 (17.6%) overlapped annotated genes in the *P. vulgaris* reference genome, respectively (Figure 3A). The largest SV was an apparent deletion of 3325 bp affecting an entire intron of the *P. vulgaris* gene Phvul.005G156800. This region was assembled with a consistently different structure in the Midas cultivar, thus also influencing gene annotation and revealing a structure not identifiable in the *P. vulgaris* reference genome (Figure 3A–C). We observed clusters of SNVs and indels overlapping either coding sequences or introns (Figure S3), affecting both the nucleotide and protein sequence of orthologous genes in the *P. vulgaris* reference genome and Midas cultivar, as demonstrated by the <100% identity between the alignments (Figure 4A). To determine whether the Cas9-based reconstruction could improve variant calling, we aligned Midas WGS Illumina data to the *P. vulgaris* reference genome after replacing the ROI with the Cas9 assembly (Figure S1B). A greater number of Illumina WGS reads mapped to genes annotated on the Cas9 assembly compared to orthologs on the *P. vulgaris* reference genome (+9.18% ± 6.74, mean ± SEM) (Figure 4B). The genotypability (% PASS at ≥5 reads) also increased for six of the 30 genes (Figure 4C).

### 2.2. Validation of the De Novo Assembly Generated by Cas9 Tiling Using a Traditional WGS Approach

**Figure 5:**
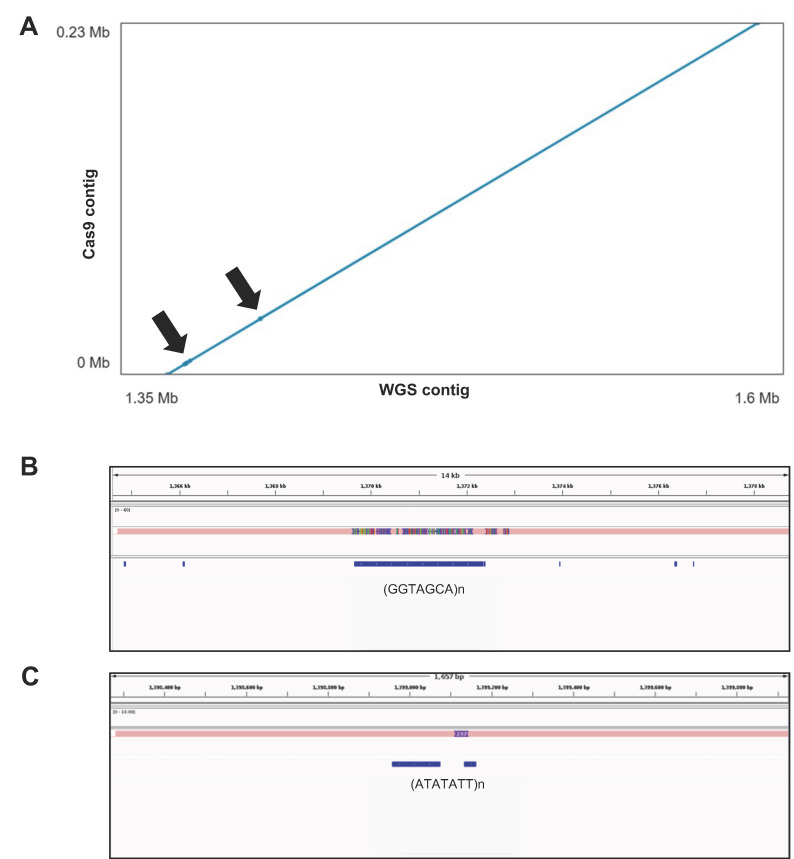
Comparison of de novo assemblies from Cas9 tiling and nanopore WGS data in the pod-shattering region. (**A**) Dot plot viewer of contig–contig alignment showing the nanopore WGS assembly on the *x*-axis and the Cas9 assembly on the *y*-axis. Divergent regions are highlighted with arrows. (**B**,**C**) Integrative Genome Viewer (IGV) visualization of the aligned Cas9-derived and nanopore WGS-derived contigs at the divergent regions. Blue boxes indicate repetitive regions annotated by RepeatMasker.

The accuracy of the ROI assembly generated by Cas9 tiling was confirmed by traditional whole genome de novo assembly in parallel (Figure S1A). ONT-based WGS of the Midas cultivar produced 992,031 PASS reads (78% of the total) with an N50 value of 43 kbp, corresponding to an equivalent average coverage of 58× (Table 2). The corresponding de novo assembly generated a genome of 509.2 Mbp comprising 1913 contigs with an N50 value of 3.4 Mb, which was polished using Midas Illumina WGS data. The size of the contig containing the ROI was 3.7 Mbp (Table 3) and showed very high structural and sequence concordance with the region reconstructed by Cas9 tiling (Figure 5A). The two sequences showed 99.5% alignment identity and featured only two regions (2.7 kbp and 300 bp, respectively) with multiple differences (Figure 5A). Manual inspection revealed the presence of low-complexity and homopolymeric stretches in these regions, which are known to generate errors during ONT sequencing (Figure 5B,C).

## 3. Discussion

We evaluated the accuracy of a targeted de novo assembly strategy based on Cas9 tiling for the reconstruction a large ROI (250 kbp), namely the pod-shattering locus in *P. vulgaris*. The Cas9 tiling approach allowed the reconstruction of the entire region as a single contig, showing high concordance with the corresponding assembly based on traditional long-read WGS. The reconstructed region improved read mapping onto annotated genes, increasing the accuracy of variant calling. This avoided dependence on the *P. vulgaris* reference genome, which diverged significantly from the Midas cultivar in sequence and structure within this region and would, therefore, introduce biases, mapping artifacts, and incorrect/missing variants due to non-mapping reads.

Sequencing costs continue to decline, making the de novo assembly of reference genomes more accessible for several species, including plants. However, such genomes are frequently reconstructed using a semi-automatic process, leaving parts of the assembly incomplete or imprecise and reducing the accuracy of downstream analysis. The Cas9 tiling method described herein can improve the reconstruction of large but confined genomic ROIs. This is particularly beneficial in the case of plants with large and complex genomes, which limit the ability to generate multiple whole-genome assemblies in a large set of individuals. The reconstructed ROI can replace poor-quality assemblies in the reference genome and can fill gaps, as already attempted in the Japanese plum (*Prunus salicina*) [22]. We found that the availability of more precise reconstructions allows reads to be mapped more accurately in cultivars/varieties of interest and facilitates downstream variant calling. As such, the approach can be exploited for GWAS or the fine mapping of ROIs associated with a given phenotype. By integrating the region reconstructed by Cas9 tiling into the reference genome, short-read sequencing data from a large number of inbred individuals can be analyzed to achieve more accurate genotyping.

Alternatively, the Cas9-mediated sequencing can be used to directly analyze SVs and tandem repeats, taking advantage of long-reads. This can allow the reconstruction of pangenomic ROIs to unravel genetic diversity associated with a phenotype of interest at the sequence and structural levels in large sets of individuals. Moreover, the same Cas9 sequencing data can help to identify SNVs and indels in downstream genotype–phenotype association studies, or to develop trait-associated SNV markers for marker-assisted selection. This has already been attempted in Japanese plum and apple [22,23], albeit with limited accuracy, but the newly released ONT Q20+ chemistry is expected to improve this aspect. Finally, thanks to the direct sequencing of native DNA, ONT also provides an opportunity to measure the level of DNA methylation in the ROI, which can help to decipher gene expression programs and their impact on phenotype.

From a technical perspective, Cas9 tiling subdivides the ROI into smaller overlapping parts, thus facilitating subsequent de novo assembly. Although the Cas9 system has already been used for de novo assembly, these previous studies either involved consistently shorter ROIs (8 kbp) or did not reconstruct the whole ROI [22,23]. Most importantly, the accuracy of the reconstructions was never confirmed. Here, we showed there was no significant sequence or structural variation between contigs assembled from ONT data by WGS or Cas9-mediated target enrichment, making the approaches interchangeable. The only two discrepancies coincided with two regions featuring low-complexity and homopolymeric stretches, where the ONT sequencing chemistry we used (R9.4.1) is known to be error-prone. Although we cannot exclude the possibility of errors in either assembly, Cas9 data are likely to produce more accurate reconstructions because a two-fold higher degree of target coverage was achieved compared to WGS. Furthermore, polishing using short reads may have suffered from low mapping quality issues in these regions, thus failing to achieve proper assembly correction. Given the dual reader head of the new Q20+ chemistry, higher sequencing accuracy is anticipated on such genomic features and should further reduce these residual errors.

ONT recommends that Cas9-mediated sequencing is limited to a maximum ROI size of 20 kbp to avoid reducing the efficiency of enrichment and sequencing. Accordingly, the only previous studies using Cas9 tiling enriched sub-ROIs of 10–25 kbp [25]. In contrast, we demonstrated that the system can enrich regions of ~50 kbp, making it more cost-effective for the targeted sequencing of very large ROIs. Cas9 has also been used to enrich and sequence targets > 100 kbp in length by applying adjustments such as target pull-down or background removal with endonucleases [24,32]. However, these approaches achieve lower target coverage depth that may be not sufficient for the subsequent de novo assembly of the target region.

To ensure the efficiently targeted sequencing of the entire 50-kbp ROI, we used a three-step purification protocol to extract high-molecular-weight (HMW) DNA, beginning with nuclear isolation, followed by gradient stratification and finally extraction on gravity-flow columns. This was necessary because ONT sequencing is inhibited by plant-derived contaminants and the sequencing yield is inversely correlated to the read length [33,34]. Plant tissues contain large quantities of secondary metabolites that co-purify with DNA, such as phenolics and polysaccharides, which interfere with library preparation and cause pore clogging [34,35,36]. HMW DNA also forms secondary structures that indirectly lead to pore clogging, particularly when derived from plant genomes that are rich in repetitive regions. All these factors can limit the output of ONT flow cells and hinder the sequencing of very long DNA fragments. The HMW DNA extraction method we used produces consistently longer reads compared to other Cas9-mediated sequencing experiments in plants [22,23], thus encompassing the whole ROI without compromising the coverage. Furthermore, although ONT flow cells can sequence DNA fragments several Mbp in length, short fragments can outcompete longer ones because they are sequenced more quickly. The Cas9-mediated sequencing method can, therefore, be improved by processing the starting DNA using the Short Read Eliminator kit (Circulomics) or the Short Fragment Eliminator kit (ONT), which selectively precipitate and exclude shorter fragments, increasing the average read length.

Finally, the outcome of Cas9-targeted sequencing is also strongly influenced by the cutting efficiency of Cas9 at particular gRNA target sites. We, therefore, designed all gRNAs to target coding regions, which are generally more conserved than intergenic and intronic sequences. We also used the available Illumina WGS data to avoid regions with SNVs and indels in the target sequences. If such data are unavailable, publicly available WGS or RNA-Seq data can be used instead. To reduce the risk of failure and to maximize coverage, two or more gRNAs can be designed to flank the sub-ROIs and can be used in combination or individually and then pooled for DNA enrichment and sequencing.

In conclusion, we have demonstrated that Cas9 tiling allows the efficient de novo reconstruction of very large target regions and produces high-quality assemblies. This allows us to generate improved assemblies featuring more accurate sequences and structures, which can be used for downstream population studies requiring genotyping and the analysis of structural variation.

## 4. Materials and Methods

### 4.1. Extraction of HMW DNA

HMW DNA was extracted from young leaves of *P. vulgaris* cv. Midas plants freshly collected after incubation for 24 h in the dark. Nuclei were isolated as previously described with minor modifications. Briefly, frozen leaves were ground to powder and mixed with 45 mL of freshly prepared MEB buffer comprising 1 M 2-methyl-2,4-pentanediol (MPD), 10 mM piperazine-*N*,*N*′-bis(2-ethanesulfonic acid) (PIPES)-KOH, 10 mM MgCl_2_, 2% polyvinylpyrrolidone (PVP)-10, 10 mM sodium metabisulfite, 0.5% sodium diethyldithiocarbamate, 6 mM ethylene glycol-bis(β-aminoethyl ether)-*N*,*N*,*N*′,*N*′-tetraacetic acid (EGTA), 200 mM L-lysine-HCl, and 5 mM β-mercaptoethanol (pH 5). The homogenate was filtered through 100 μm and 40 μm cell strainers and mixed with 0.5% Triton X-100. After incubation on ice for 30 min, the homogenate was centrifuged (800× *g*, 20 min, 4 °C) and the pellet was re-suspended in 45 mL of MPDB buffer (0.5 M MPD, 10 mM PIPES-KOH, 10 mM MgCl_2_, 0.5% Triton X-100, 10 mM sodium metabisulfite, 5 mM β-mercaptoethanol, pH 7.0). After up to four rounds of centrifugation as above, in each case saving the whiter layer of the pellet representing the nuclei, the white pellet was layered onto a 20-mL 37.5% Percoll bed in MPDB buffer in a 15-mL glass centrifuge tube. The gradient was centrifuged twice (650× *g*, 1 h, 4 °C) and the white part of the pellet was recovered and resuspended in 10 mL MPDB buffer before two further rounds of centrifugation (2500× *g*, 10 min, 4 °C). The supernatant was discarded and HMW DNA was isolated using adsorption-based gravity columns QIAGEN Genomic tip (QIAGEN, Hilden, Germany). The quantity of DNA was assessed using a Qubit fluorometer and dsDNA broad-range assay kit (both from Thermo Fisher Scientific, Waltham, MA, USA). The size of the HMW DNA was assessed by pulsed-field gel electrophoresis (PFGE).

### 4.2. Illumina Sequencing and Data Analysis

We fragmented 1 μg of genomic DNA using an S220 sonicator (Covaris, Woburn, MA, USA) to achieve an average size of 400 bp. Illumina PCR-free libraries were prepared starting from 1 μg of fragmented DNA and unique dual-indexed adapters (5 μL of a 15 μM stock) using the KAPA Hyper prep protocol (Kappa Biosystems, Basel, Switzerland) with minor modifications. These included increasing the adapter ligation time to 30 min and introducing a post-cleanup size selection step using 0.7× AMPure XP beads (Beckman Coulter, Brea, CA, USA). The library concentration and size distribution were assessed on a Bioanalyzer 2100 with high-sensitivity DNA reagents/chips (all from Agilent Technologies, Santa Clara, CA, USA). Sequencing was carried out on a NovaSeq6000 instrument (Illumina, San Diego, CA, USA) to generate 150-bp paired-end reads.

Trimmed Illumina reads were aligned to the genome using BWA mem v0.7.17 [37]. Duplicates were marked and removed with Picard MarkDuplicates v2.18.29 [38]. Overlapping reads from the same fragment were then hard-clipped with fgbio ClipBam v1.3.0 [39]. SNVs and indels were called using GATK HaplotypeCaller v4.2.0.0 and filtered using the SelectVariants and VariantFiltration routines [40]. The percentage of callable bases for each region of interest has been calculated with GATK CallableLoci v3.8 [40] using “-minDepth 5” and modifying genomic coordinates (“-L” parameter) accordingly.

### 4.3. Cas9 Tiling Coupled to ONT Sequencing

CRISPR RNAs (crRNAs) were manually designed by selecting crRNA target regions in the reference genome (*P. vulgaris*_442_v2.0) that were annotated with genes and well covered by Illumina WGS reads from the Midas cultivar. All possible candidate crRNAs were designed on the selected regions using the Custom Alt-R CRISPR-Cas9 guide RNA online tool from Integrated DNA Technologies (IDT, Coralville, IA, USA) [41] and by checking the “other species” option. To verify off-targets, candidate gRNAs were aligned to the reference genome using BLAST, and crRNAs with only one target were retained. Illumina WGS reads from the Midas cultivar were also used to further select crRNAs that did not overlap with SNVs or indels. Based on these criteria, we designed 10 crRNAs to enrich sub-ROIs with an average length of 49.8 kbp, resulting in five sub-ROIs with overlaps of ~2–3 kbp that covered the ROI. The crRNAs were synthesized at the 2 nmol scale by IDT (Table 1).

Enrichment by Cas9 tiling was performed according to the ONT’s targeted, amplification-free DNA sequencing using CRISPR/Cas protocol (v.ENR_9084_v109_revD_04Dec2018). The crRNAs were divided into two pools so that P1 would excise sub-ROIs 1, 3 and 5 and P2 would excise sub-ROIs 2 and 4. Two separate mixtures of six (P1) and four (P2) crRNAs (10 μM each) were prepared and each mixture was completed by adding 10 μM transactivation crRNA (tracrRNA) and duplex buffer (both from IDT) and then denatured at 95 °C for 5 min and cooled to room temperature for 10 min. Ribonucleoproteins (RNPs) representing P1 and P2 were formed by mixing each 10 μM gRNA pool with 62 μM Alt-R S.p. HiFi Cas9 nuclease V3 (IDT) in 1× CutSmart Buffer (New England Biolabs, Ipswich, MA, USA) and incubating for 30 min at room temperature. Each pool was used to cut 5 μg of HMW DNA. At the end of the procedure, the two reactions were pooled and the mixture was purified using 0.3× AMPure XP magnetic beads. The beads were washed twice with 250 μL Long Fragment Buffer (ONT) to remove short fragments (<3 kbp) and DNA was eluted for 10 min at room temperature using 13 μL of Elution Buffer (ONT). The final Cas9 library was sequenced on a MinION device (FLO-MIN106D, R9.4.1) using MinKNOW v20.06.5 (ONT) until a plateau in data production was observed.

### 4.4. Nanopore WGS

We processed 10 µg of HMW DNA using the short read eliminator kit (PacBio, Menlo Park, CA, USA) to remove fragments < 25 kbp. Two libraries were prepared from two aliquots (4 µg each) of genomic DNA without initial fragmentation, following ONT’s genomic DNA by ligation protocol (v.GDE_9063_v109_revAE_14Aug2019). Nanopore sequencing was carried out on a MinION device (FLO-MIN106D, R9.4.1) using MinKNOW v20.06.5. Each time a run reached saturation; the flow cell was washed using ONT’s flow cell wash kit (EXP-WSH002). Flow cells 1 and 2 were loaded three times and twice, respectively. All data were merged before whole-genome de novo assembly.

### 4.5. ONT Sequence Analysis and De Novo Assembly

ONT data were collected as fast5 files and basecalling was carried out using Guppy v4.0.11 in high-accuracy mode. Reads were then quality filtered using NanoFilt v2.7.1 [42], retaining all reads with Q-score > 7.

Nanopore WGS reads were first corrected using Canu v2.0 [43] with default parameters, setting genomeSize to 500 Mb. Corrected reads were then assembled de novo using Wtbdg v2.5 [44] with default parameters. A first round of polishing was carried out by mapping nanopore reads to the draft genome assembly with Minimap2 v2.21-r1071 [45] and running a combination of Racon v1.5.0 [46] and Medaka v1.6.0 [47]. A second round of polishing was then carried out by mapping Illumina reads to the polished assembly using BWA mem v0.7.17 [37] and running Pilon v1.23 [48] with default parameters.

Nanopore reads from Cas9 tiling were aligned to the *P. vulgaris* v2.1 reference genome using Minimap2 v2.21-r1071 [45], and only reads overlapping the ROI were extracted using samtools v1.19 [49]. On-target reads were assembled de novo using Canu v2.0 [43] with default parameters, setting genomeSize to 250 kbp. The first round of polishing was carried out as described above for the WGS reads. The assembled contig was then inserted into the *P. vulgaris* v2.1 genome, replacing the corresponding region of the genome assembly. This allowed us to reduce spurious alignments of Illumina WGS reads to a single contig. A second round of polishing was then carried out by mapping Illumina reads to the *P. vulgaris* v2.1 genome assembly with the inserted contig using BWA mem v0.7.17 [37] and running Pilon v1.23 [48] with default parameters. The polished contig was excised from the *P. vulgaris* v2.1 genome.

### 4.6. Comparison of Cas9-Tiling Assembly with P. vulgaris Reference Genome and WGS Assembly

The contig obtained based on Cas9 tiling data was mapped to the whole genome assembly with Minimap2 v2.21-r1071 using “-x map-ont” parameter [45]. The resulting sam file was converted to delta format using samtodelta.py script available from https://github.com/malonge/RaGOO (accessed on 10 August 2022) and, finally, uploaded to Assemblytics webpage (http://assemblytics.com/, accessed on 10 August 2022) [50] to identify insertions and deletions. The percentage of identity was then quantified as a ratio between non-matching characters (NM tag) and alignment length, after extracting corresponding information from the CIGAR string. To identify single nucleotide differences, the contig obtained by Cas9 tiling was aligned to the whole-genome assembly using the nucmer subroutine of MUMmer v4.0.0beta2 [51] and the variants identified using nucdiff [52]. For visualization purposes, alignment was visualized using dot, an interactive dot plot viewer for genome–genome alignments [53]. Predicted CDS and gene sequences (pre-mRNA loci excluding UTR regions) were compared with *P. vulgaris* official annotation using blast version 2.9.0+ software.

### 4.7. Genome Annotation

Protein coding genes were annotated on the *P. vulgaris* reference genome following the insertion of the Cas9-derived contig. Before gene prediction, repetitive sequences were identified and genomes were soft-masked using RepeatMasker v4.1.2-p1 [54]. Repeats were identified by constructing a custom repeat library using RepeatModeler v2.0.2 [55] with the LTR module. Genes were identified using the Augustus v3.3.3 [56] ab initio predictor in hints mode, with the external evidence comprising proteins of *P. vulgaris*, barrel clover (*Medicago truncatula*) and soybean (*Glycine max*) to identify coding sequences and introns. Protein data were aligned using Genome Threader v1.7.1 [57] with parameters mincoverage 0.65 and minalignmentscore 0.7. We also integrated 21 RNA-Seq datasets representing domesticated and wild accessions (unpublished data and from [58]), which were trimmed using an internal custom script and aligned to PAV regions using HISAT2 v2-2.2.1 [59] with parameter —max-intronlen 23,000 -k 1. The aligned data were converted into intronic hints, which were removed if fewer than 20 split reads were available as support. BUSCO [60] genes contained in the Fabales_odb10 database were used to train the gene predictor models on the assembled genome (BUSCO v4.1.4). Only genes intersecting the Cas9 tiling region were retained for downstream analysis. Gene models were manually curated using the aligned RNA-Seq data, and gene models without supporting RNA-Seq data were excluded from the comparison with *P. vulgaris* orthologs. Untranslated regions were also excluded because they were not predicted in the de novo annotation.

## Figures and Tables

**Table 1:** CRISPR RNAs used in the study. The crRNA ID, sequence and position of the target in the *P. vulgaris* reference genome are shown, and The crRNAs are grouped according to the sub-ROI they cut and the reaction pool (P1 or P2).

ID	Sequence crRNA	PAM	Chr	[Start]	[End]	SubROI	SubROI Length (kbp)
gRNA.1	CTCAAGGGTCGTAACATTCC	TGG	5	38,489,481	38,489,500	SubROI1_P1	54.4
gRNA.2	TATGATGACACACACGTTAA	CGG	5	38,540,913	38,540,894		
gRNA.3	ATGCCATTAAGAGTTGCGAT	GGG	5	38,537,240	38,537,259	SubROI2_P2	45
gRNA.4	TTTTCACGACTTTGCATCTT	TGG	5	38,582,350	38,582,331		
gRNA.5	AGAACGGAAGGAATGGGACA	GGG	5	38,580,180	38,580,199	SubROI3_P1	48.6
gRNA.6	GGATATTACAAACAGACGAA	AGG	5	38,628,876	38,628,857		
gRNA.7	ACTGTTGCGTAGGGACAAAT	CGG	5	38,626,429	38,626,448	SubROI4_P2	48.3
gRNA.8	AGTTTGACAACTATCCCAAG	GGG	5	38,674,838	38,674,819		
gRNA.9	GCCACTATAGTGCCAACTTC	TGG	5	38,671,239	38,671,258	SubROI5_P1	52.5
gRNA.10	ATTACCGTAGCTAGTTATTA	AGG	5	38,723,776	38,723,757		

**Table 2:** ONT sequencing statistics, comparing Cas9 tiling and whole genome sequencing (WGS).

	Cas9 Tiling	WGS
Sequencing output (Gbp)	0.84	32.00
Total reads	157,028	1,246,133
Total aligned PASS reads	54,540	992,031
PASS read N50 (bp)	30,121	43,366
On-target PASS reads on ROI	1794	375
On-target reads %	3.29%	0.04%
On-target reads fully spanning sub-ROIs	143	13
On-target average coverage	152.85×	67.32×
Whole genome average coverage	1.21×	43.63×
Fold enrichment	113.16×	1.36×

**Table 3:** ONT assembly statistics, comparing Cas9 tiling and whole genome sequencing (WGS).

	Cas9 Tiling	WGS
Total assembly length (bp)	229,302	509,180,482
Number of contigs	1	1913
Contig N50 (bp)	229,302	3,412,857
Contig average length (bp)	229,302	266,169
Contig including ROI (bp)	229,302	3,708,722

## Data Availability

The sequencing data generated in this study have been submitted to NCBI GenBank (BioProject PRJNA905799) under accession numbers.

## Supplementary Materials

The following supporting information can be downloaded at: https://www.mdpi.com/article/10.3390/ijms24021076/s1.
