# Supplementary material for: CRISPR/Cas9-Mediated Enrichment Coupled to Nanopore Sequencing Provides a Valuable Tool for the Precise Reconstruction of Large Genomic Target Regions
Source: Int J Mol Sci. 2023 Jan 5;24(2):1076. doi: 10.3390/ijms24021076 (PMC9863143; doi:10.3390/ijms24021076)

CRISPR/Cas9-Mediated Enrichment Coupled to Nanopore Sequencing Provides a Valuable Tool for the Precise Reconstruction of Large Genomic Target Regions

Giulia Lopatriello [1,†], Simone Maestri [1,†], Massimiliano Alfano [1], Roberto Papa [2], Valerio Di Vittori [2], Luca De Antoni [1], Elisa Bellucci [2], Alice Pieri [2], Elena Bitocchi [2], Massimo Delledonne [1,3,*] and Marzia Rossato [1,3,*]

1 Department of Biotechnology, University of Verona, Strada Le Grazie 15, 37134 Verona, Italy
2 Department of Agricultural, Food and Environmental Sciences, Polytechnic University of Marche, via Brecce Bianche, 60131 Ancona, Italy
3 Genartis srl, Via IV Novembre 24, 37126 Verona, Italy
* Correspondence: massimo.delledonne@univr.it (M.D.); marzia.rossato@univr.it (M.R.); Tel.: +39-0458027800 (M.R.)
† These authors contributed equally to this work.

Supplementary Material

Figure S1. Workflow of analysis. (A) *De novo* assembly workflow utilized in the study to reconstruct the shattering-associated region in the Midas cultivar, either using the Cas9-tiling or the traditional WGS approach. The assembly generated from Cas9-tiling data was compared with the ROI of the *P. vulgaris* reference genome and the WGS-derived assembly. **(B)** Illumina WGS data were mapped either on the *P. vulgaris* reference genome or on the *P. vulgaris* reference where the Cas9-assembly replaced the ROI. Number of read mapping and genotypability were evaluated in the whole ROI and on genes present in the ROI.

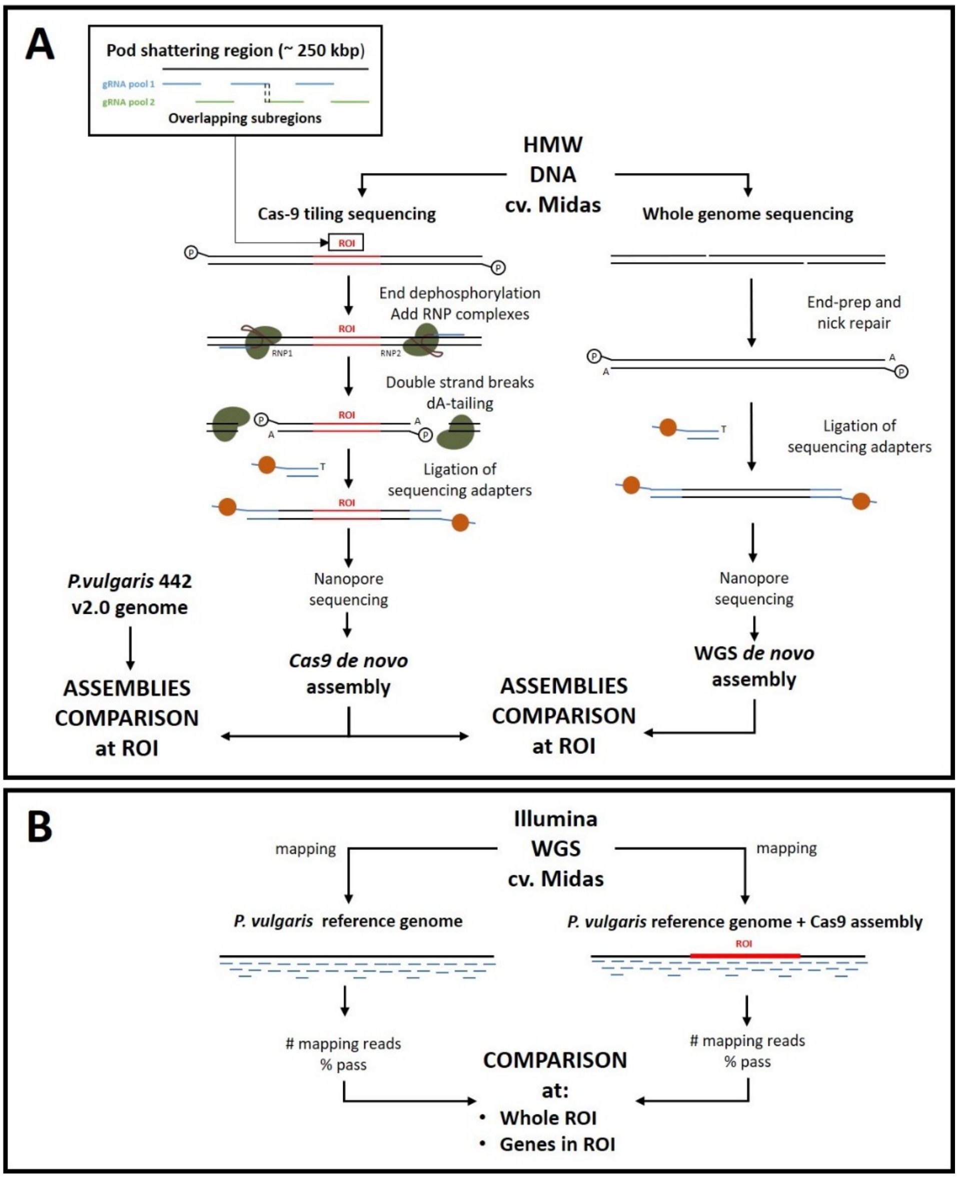

Figure S2. Design of gRNAs for Cas9 tiling of the pod-shattering region. Integrative Genome Browser Visualization (IGV) of the pod-shattering region showing the tracks of the five overlapping sub-ROIs cut by pairs of flanking Cas9-gRNA complexes. The protospacer sequences were designed to target genomic regions annotated with genes (red tracks). Sub-ROIs 1, 3, 5 (green tracks), 2 and 4 (blue tracks) were enriched in two parallel reactions.

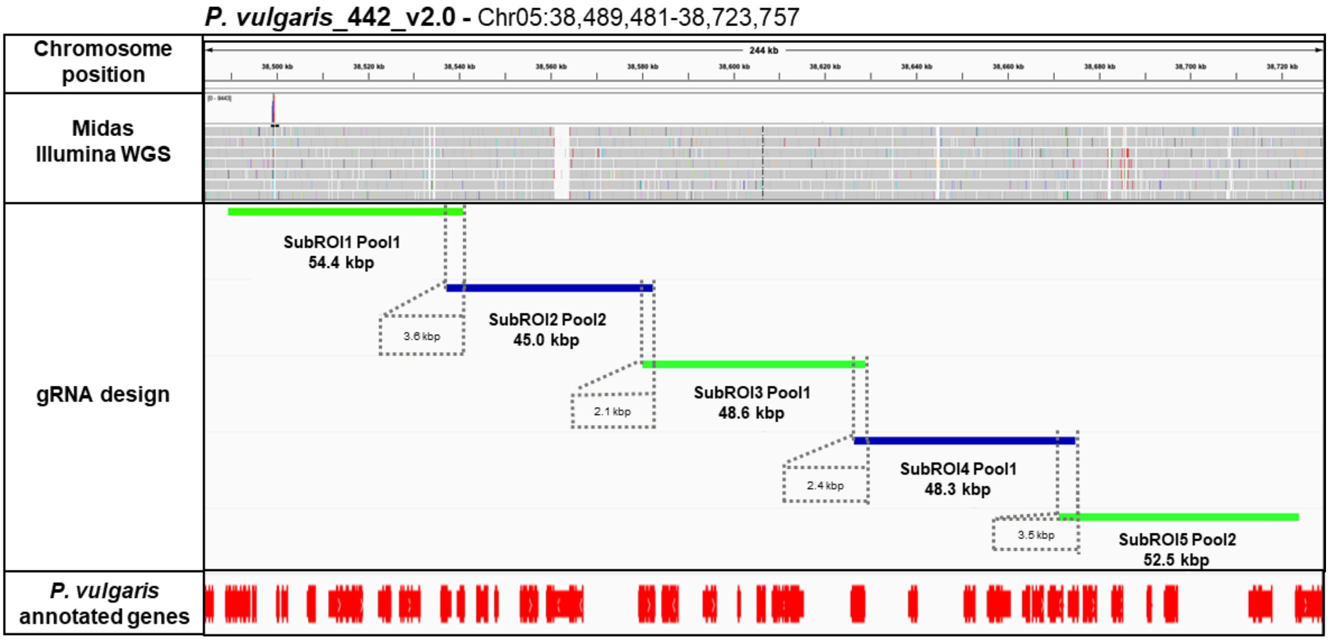

Figure S3. The pod-shattering region features a large number of variations, some of which overlap genes, when comparing the Midas cultivar and *P. vulgaris* reference genome. (A-B) Integrative Genome Browser Visualization (IGV) of the Cas9 assembly and Midas Illumina WGS reads aligned to the *P. vulgaris* reference genome revealing the detailed tracks of SNVs and SVs identified in the Cas9 assembly compared to the reference genome and the annotated genes (Phvul.005G156200.1.p and Phvul.005G158300.1.p, respectively). **(C-D)** IGV of Midas Illumina WGS reads aligned to the Cas9 assembly in the region including the orthologous gene shown in panels A and B.

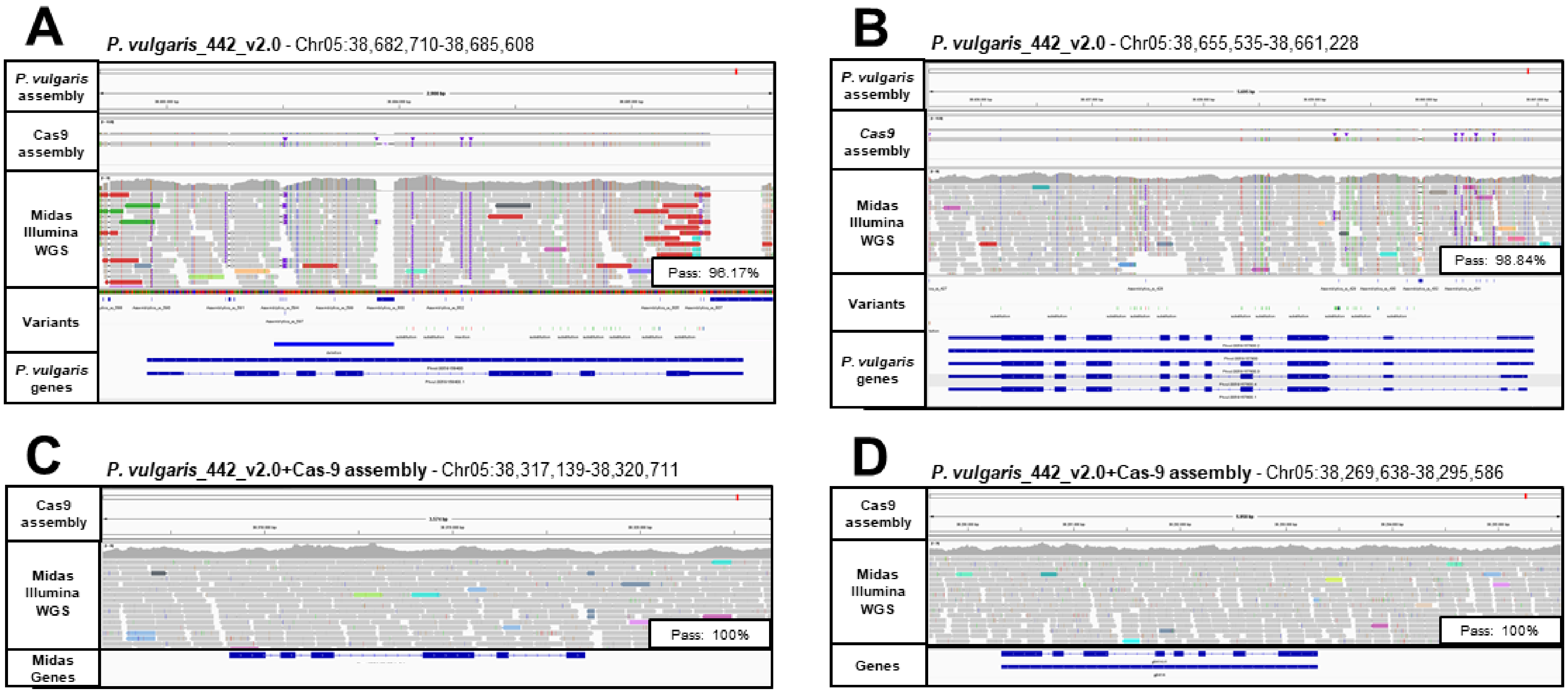

Supplement: Supplementary file 1 [file ijms-24-01076-s001.zip › ijms-2102279-supplementary.pdf]
